# Incisional hernia after surgery for colorectal cancer: a population-based register study

**DOI:** 10.1007/s00384-018-3124-5

**Published:** 2018-07-17

**Authors:** Harald Söderbäck, Ulf Gunnarsson, Per Hellman, Gabriel Sandblom

**Affiliations:** 1 Department of Surgery, Capio St Göran Hospital, Stockholm, Sweden; 2 Department of Clinical Science Intervention and Technology (CLINTEC), Karolinska Institutet, Stockholm, Sweden; 3 Department of Surgical and Perioperative Sciences, Umeå University, SE-901 87 Umeå, Sweden; 4 Department of Surgical Sciences, Uppsala University, SE-751 85 Uppsala, Sweden; 5 Department of Clinical Science and Education, Södersjukhuset, Karolinska Institutet, Stockholm, Sweden; 6 Department of Surgery, Södersjukhuset, Stockholm, Sweden

**Keywords:** Incisional hernia, Colorectal cancer, Risk factors

## Abstract

**Background:**

Our knowledge on the incidence of incisional hernia and risk factors for developing incisional hernia following surgery for colorectal cancer is far from complete.

**Methods:**

All procedures registered in the Swedish Colorectal Cancer Register (SCRCR) 2007–2013 were identified. Patients with comorbid disease diagnoses, registered at admissions and visits prior to the procedure and relevant to this study, were obtained from the National Patient Register (NPR). These diagnoses included cardiovascular disease, connective tissue disorders, liver cirrhosis, renal failure, diabetes, chronic obstructive lung disease and chronic inflammatory conditions. Data on occurrence of incisional hernias were obtained by combining data from the SCRCR and the NPR (International Classification of Diseases code).

**Results:**

During 2007–2013, 39,984 procedures were registered in the SCRCR. After excluding laparoscopic procedures, procedures repeated on the same patient, procedures with concomitant liver resection and procedures without laparotomy, 28,913 cases remained for analysis. Five years after surgery, the cumulative incidence of incisional hernia was 5.3%. In multivariate proportional hazard analysis, significantly increased risk for incisional hernia was found for the male gender (hazard ratio [HR] 1.40, 95% confidence interval [CI] 1.21–1.62), operation time exceeding 180 min (HR 1.25, CI 1.08–1.45), body mass index (BMI) > 30 (HR 1.78, CI 1.51–2.09), age < 70 years (HR 1.34, CI 1.16–1.56) and postoperative wound complication (HR 2.09, CI 1.70–2.58).

**Discussion:**

Men, patients younger than 70 years and patients with BMI > 30 face a higher risk for incisional hernia. The risk is also increased in cases where the procedure takes longer than 3 h or where postoperative wound complications occur. These patients will benefit from measures aimed at preventing the development of incisional hernia.

## Background

Incisional hernia is a potentially life-threatening and costly complication after laparotomy, with a cumulative incidence in studies of up to 20% [1]. The impact of incisional hernia on health-related quality of life and physical function is often underestimated since the hernia may not be clinically obvious until several years after the operation and thus does not always come to the attention of the surgeon. It may thus be possible that the risk for incisional hernia may be neglected when deciding on surgery due to lack of awareness of factors that increase this risk [2, 3].

A small rate of incisional hernia after abdominal surgery has always been considered inevitable and a problem that must be weighed against the potential benefit gained from the index procedure. As a result, our efforts have previously been concentrated more on treatment and recurrence of incisional hernia in patients developing an abdominal wall defect than on exploring possible ways to prevent these hernias from occurring. This defeatist view, however, is now changing towards a more active approach regarding hernia prevention and the optimisation of methods used for abdominal wall closure at the index procedure.

Recent studies have shown that it is possible to reduce the risk for wound dehiscence and incisional hernia by using appropriate and meticulous suture technique. However, even with meticulous sutures, approximately 5% of patients undergoing laparotomy still suffer from wound complication [4]. The aims of the present study were to estimate the risk for occurrence of incisional hernia and to explore the extent to which the presence of comorbidity influences the risk for incisional hernia.

## Methods

All procedures for colorectal cancer registered in the Swedish Colorectal Cancer Register (SCRCR) [http://www.cancercentrum.se/samverkan/cancerdiagnoser/tjock--och-ändtarm/kvalitetsregister/] 2007–2013 were identified. The SCRCR collects data from all patients diagnosed with rectal cancer since 1995 and all colon cancer patients since 2007. The Register includes data on age, gender, ASA classification, treatment and postoperative follow-up, but not data on comorbidity and pharmaceutical treatment [http://www.cancercentrum.se/samverkan/cancerdiagnoser/tjock--och-ändtarm/kvalitetsregister/]. The National Patient Register (NPR) [http://www.socialstyrelsen.se/register/halsodataregister/patientregistret/inenglish], supervised by the National Board of Health and Welfare, includes data on all hospital admissions in Sweden since 1987. It also contains data on diagnoses of patients treated in outpatient specialist care and outpatient emergency care. It does not, however, contain data on primary healthcare visits. Diagnoses from all admissions and visits prior to the colorectal cancer resection (identified by the International Classification of Diseases [ICD] code) were retrieved from the NPR in order to collect information on relevant comorbid disease. In order to identify relevant risk factors, we chose diagnoses that are considered as risk factors in clinical practice, although we could not find evidence from previous studies that they are. The diagnoses investigated were peripheral vascular disease, connective tissue disorders, liver cirrhosis, renal failure, diabetes, chronic obstructive lung disease and chronic inflammatory conditions. We also chose to analyse variables such as age, gender, operation time and preoperative radiation therapy. Cross-matching between the SCRCR and the NPR was performed in 2015 using the Swedish Personal Registration Number, a ten-digit identity number unique for each Swedish resident [5]. Data on incisional hernias were obtained by combining data obtained from the SCRCR and the NPR. The occurrence of an incisional hernia is registered in the SCRCR by the surgeon responsible. In the NPR, on the other hand, the ICD codes for incisional hernias or the intervention code for surgery for incisional hernia are recorded by the physician responsible at the time of discharge of the patient.

### Ethics statement

This study is approved by the regional ethics review board in Stockholm (ref. 2014/1351-31/5).

### Statistics

Statistical calculations were performed using SPSS 22.0 (Chicago, IL, USA). Data were retrieved in February 2015. Analyses were performed to assess the impact of each investigated risk factor and to estimate the cumulative incidence of incisional hernia. Diagnosis of incisional hernia was defined as a discharge note or outpatient visit with any of the ICD codes K43.0–K43.9. Surgery for incisional hernia was defined as the presence of any of the intervention codes JAD10–JAD87. The impact of each investigated risk factor on the incidence of incisional hernia was evaluated in a time-to-event analysis, applying the date of the primary procedure as the time of entry into the cohort. Date of death or end of follow-up was treated as a censored event. Age, body mass index (BMI), comorbid disease and type of incision as risk factors for incisional hernia were analysed using Cox proportional hazard analysis. Gender, age, BMI, history of chronic obstructive pulmonary disease, diabetes with secondary complications, chronic renal disease, liver cirrhosis, systemic inflammatory disease, T-category, distant metastases, preoperative radiotherapy, acute/planned surgery, tumour localisation, operation time, postoperative wound complication and adjuvant cytostatic treatment were included as covariates in the analysis.

## Results

**Fig. 1:**
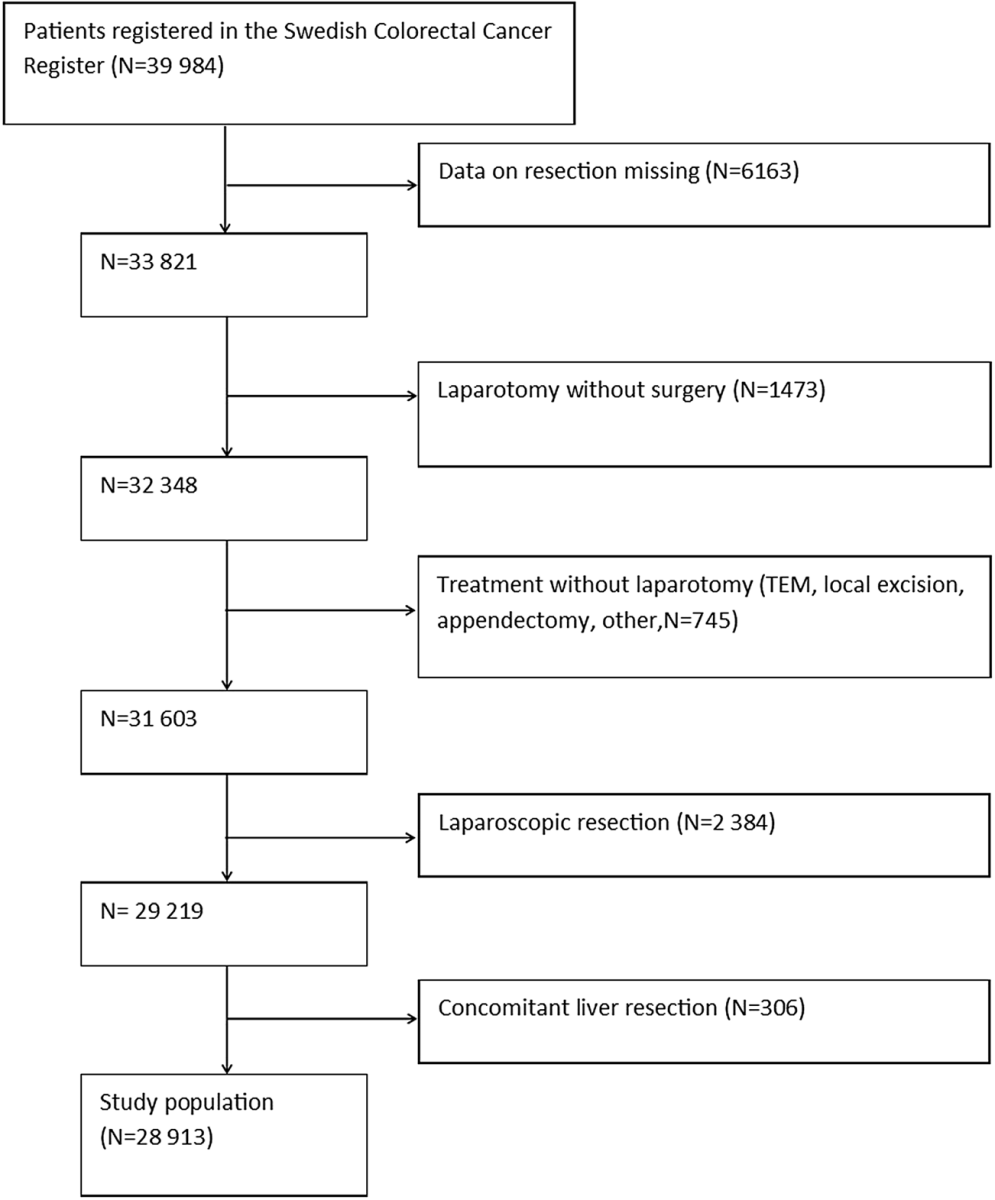
Flow chart of cohort assembly

**Fig. 2:**
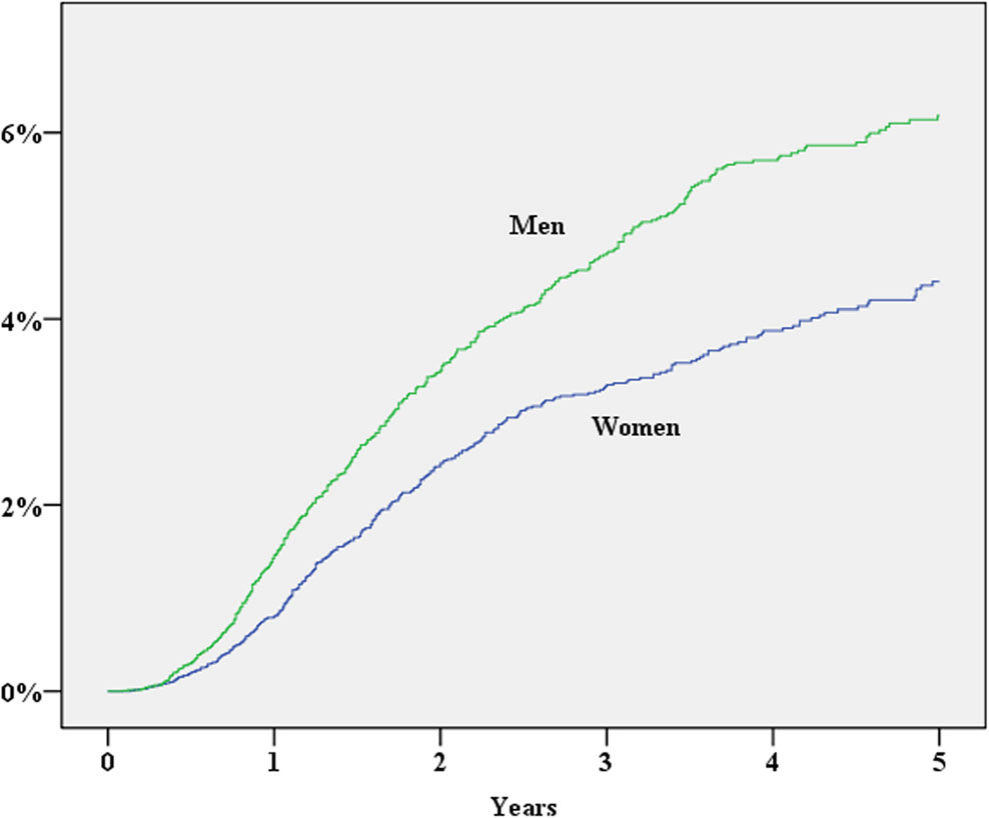
Gender vs cumulative incidence of incisional hernia

**Fig. 3:**
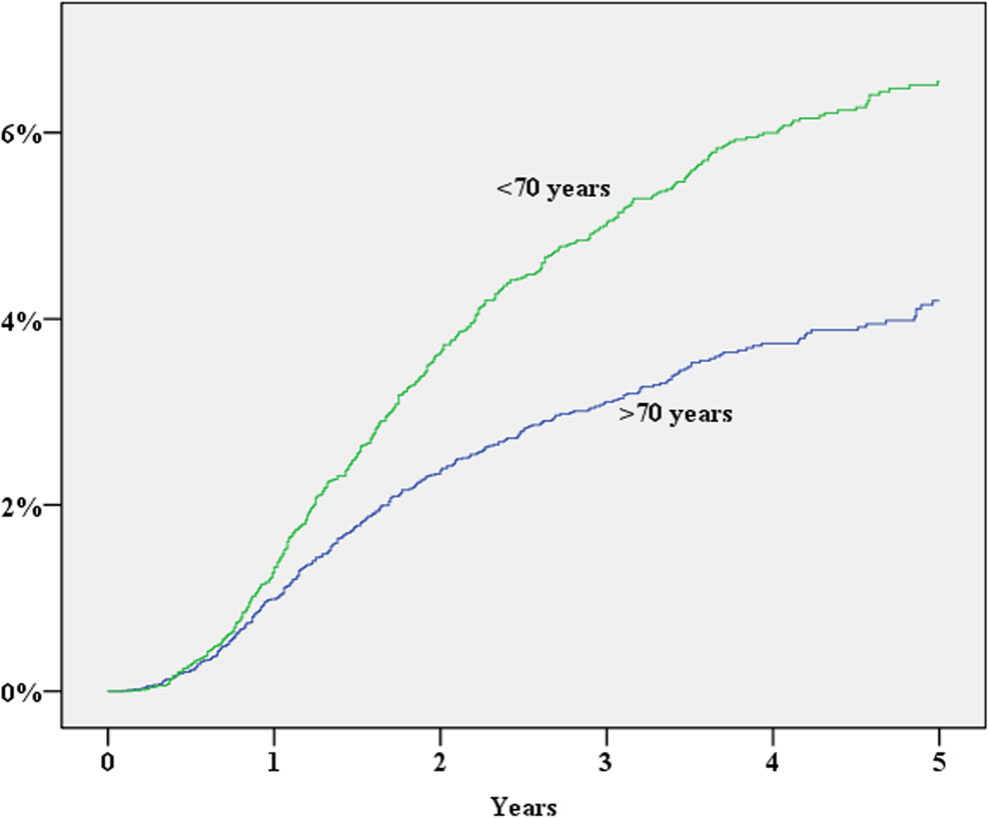
Age vs cumulative incidence of incisional hernia

**Fig. 4:**
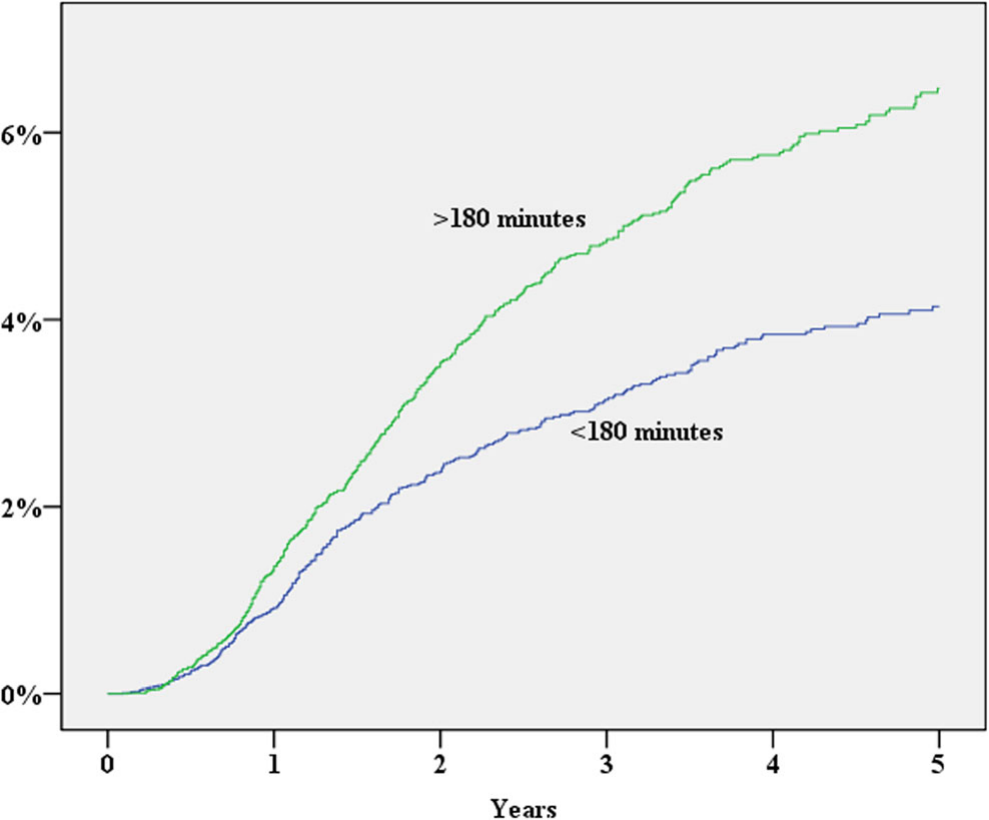
Operation time vs cumulative incidence of incisional hernia

**Fig. 5:**
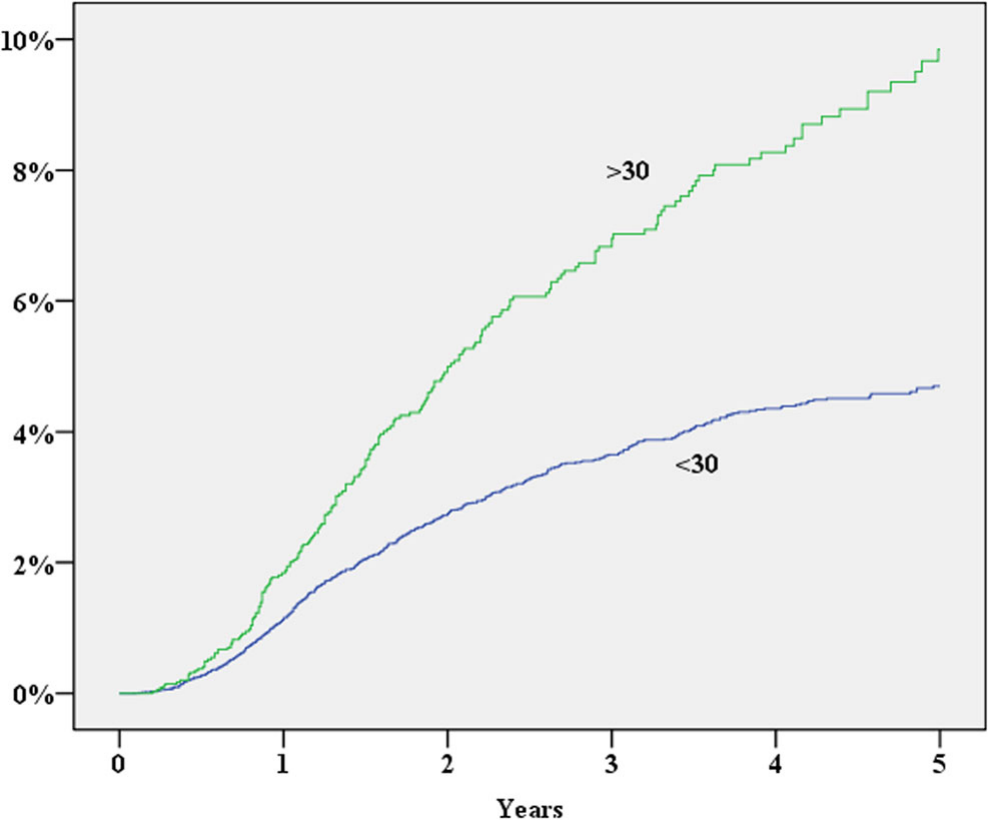
BMI vs cumulative incidence of incisional hernia

**Fig. 6:**
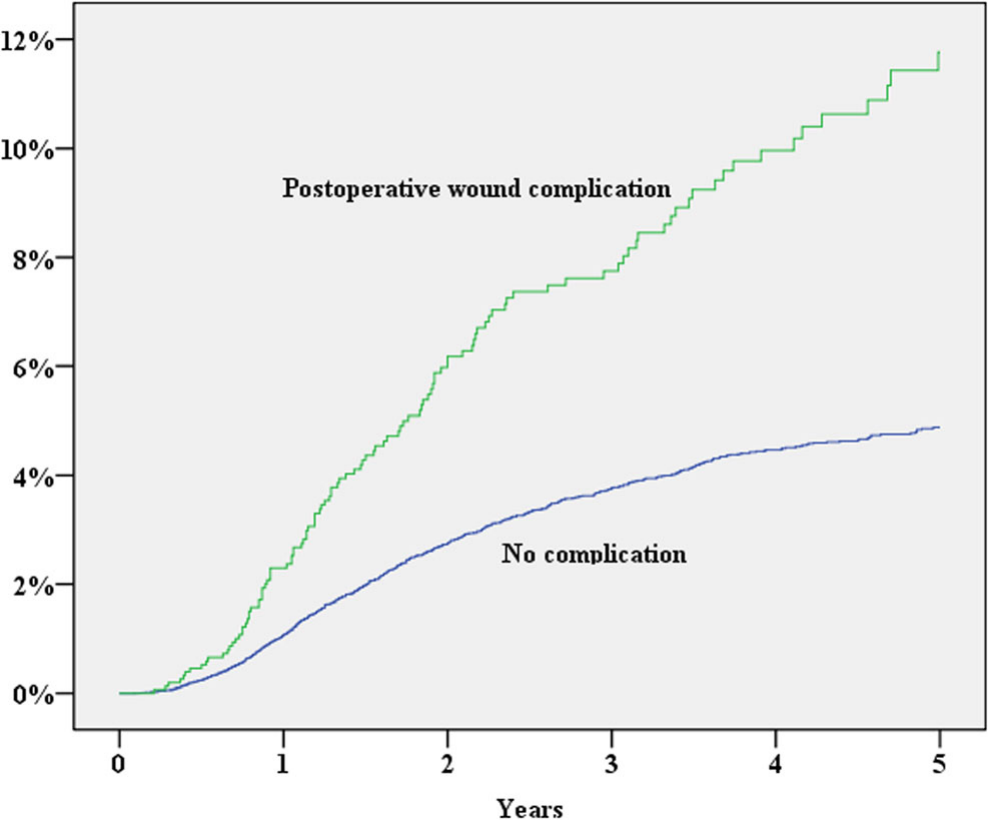
Wound complication vs cumulative incidence of incisional hernia

During 2007–2013, 39,984 patients were registered in the SCRCR. After excluding laparoscopic procedures, procedures repeated on the same patient, concomitant liver resection and procedures without laparotomy, 28,913 procedures remained for the study. The study cohort assembly flow chart is shown in Fig. 1. Baseline characteristics of the study population are shown in Table 1. During the study period, 1352 (cumulative incidence 5.3%) patients either were diagnosed with an incisional hernia or underwent surgery for this reason. In a multivariate Cox proportional hazard analysis, significantly increased risk for incisional hernia was found for the male gender, operation time exceeding 180 min, BMI > 30, age < 70 years and postoperative wound complication (Table 2; Figs. 2, 3, 4, 5, and 6). History of comorbid disease, tumour stage, tumour localisation, preoperative radiotherapy, adjuvant chemotherapy, postoperative bleeding and acute/elective surgery had no statistically significant impact.

**Table 1:** Baseline characteristics

Mean age, years (standard deviation)	71.1 (11.5)
Gender	
Male	14,986 (51.8%)
Female	13,927 (48.2%)
T	
0	292 (1.0%)
I	1585 (5.5%)
II	4595 (15.9%)
III	16,920 (58.5%)
IV?	5333 (18.4%)
TX/unknown	188 (0.6%)
N	
0	15,904 (55.0%)
I	7152 (24.7%)
II	5491 (19.0%)
NX/unknown	366 (1.2%)
M	
0	24,207 (83.7%)
I	3606 (12.5%)
MX/unknown	1200 (3.8%)
Treatment	
Resection of ascending colon (including ileocaecal resection)	10,666 (36.9%)
Resection of descending colon (including sigmoid colon)	8544 (29.6%)
Resection of rectum (anterior resection and abdominoperineal resection)	8271 (28.7%)
Other (including resection of transverse colon and total colectomy)	1432 (5.0%)
Stoma	
Temporary	4307 (15.0%)
Permanent	6083 (21.2%)

**Table 2:** Univariate and multivariate Cox proportional hazard analysis of risk for incisional hernia

		Univariate Cox proportional hazard analysis		Multivariate Cox proportional hazard analysis	
Variable	*N*	Hazard ratio (95% confidence interval)	*p*	Hazard ratio (95% confidence interval)	*p*
Gender					
Female (ref)	13,927 (48.2%)				
Male	14,986 (51.8%)	1.47 (1.29–1.67)	<0.001	1.40 (1.21–1.62)	<0.001
Age					
> 70 years (ref)	16,205 (56.0%)				
≤ 70 years	12,704 (43.9%)	1.59 (1.37–1.82)	<0.001	1.34 (1.16–1.56)	<0.001
Data on age missing	4 (<0.1%)				
BMI					
< 30	20,769 (71.8%)				
≥ 30	4036 (14.0%)	1.94 (1.65–2.22)	<0.001	1.78 (1.51–2.09)	<0.001
Data on BMI missing	4108 (14.2%)				
Chronic obstructive pulmonary disease					
No	27,600 (95.5%)				
Yes	1313 (4.5%)	1.29 (0.94–1.76)	0.112		
Complicated diabetes					
No	28,011 (96.9%)				
Yes	902 (3.1%)	0.74 (0.46–1.20)	0.225		
Chronic renal disease					
No	28,309 (97.9%)				
Yes	604 (2.1%)	0.87 (0.48–1.58)	0.656		
Liver cirrhosis					
No	28,841 (99.8%)				
Yes	72 (0.2%)	2.67 (1.08–7.67)	0.035		
Systemic inflammatory disease					
No	28,376 (98.1%)				
Yes	537 (1.9%)	1.10 (0.66–1.83)	0.725		
T					
1 (ref)	1585 (5.5%)				
0	292 (1.0%)	0.94 (0.45–1.98)	0.873		
2	4595 (15.9%)	1.13 (0.83–1.54)	0.439		
3	16,920 (58.5%)	1.01 (0.76–1.34)	0.954		
4	5333 (18.4%)	0.99 (0.71–1.40)	0.987		
TX/unknown	188 (0.7%)	1.21 (0.48–3.03)	0.684		
Distant metastases					
No (ref)	26,348 (91.1%)				
Yes	2565 (8.9%)	0.94 (0.70–1.27)	0.701		
Preoperative radiotherapy					
No	22,988 (79.5%)				
Yes	5897 (20.4%)	1.35 (1.16–1.57)	<.001		
	28 (0.1%)				
Acute/planned surgery					
Planned	24,520 (84.8%)				
Acute	4386 (15.2%)	1.04 (0.85–1.28)	0.686		
Data on acute/planned surgery	7 (<0.1%)				
Tumour localisation					
Colon	20,642 (71.4%)				
Rectum	8271 (28.6%)	1.43 (1.25–1.64)	<0.001		
Operation time					
< 180 min	13,509 (46.7%)				
≥ 180 min	14,426 (49.9%)	1.50 (1.31–1.72)	<0.001	1.25 (1.08–1.45)	0.003
Data on operation time missing	978 (3.4%)				
Postoperative wound complication					
No	27,142 (93.9%)				
Yes	1771 (6.1%)	2.29 (1.88–2.79)	<0.001	2.09 (1.70–2.58)	<0.001
Postoperative bleeding and/or transfusion					
No	28,630 (99.0%)				
Yes	283 (1.0%)	0.77 (0.35–1.73)	0.533		
Adjuvant cytostatic					
No	23,206 (80.3%)				
Yes	5707 (19.7%)	1.14 (0.97–1.34)	0.103

## Discussion

The importance of taking patient-related factors into consideration in order to identify patients with increased risk for developing a clinically significant incisional hernia was confirmed in the present study. Male patients younger than 70 years with a BMI > 30 are associated with a greater risk than patients from the rest of the population. The risk is also increased in cases where the procedure lasts longer than 3 h or where postoperative wound complications occur. By combining two large Swedish population-based registers (SCRCR and NPR), it was possible to include 28,913 operations, both acute and elective, performed nationwide in this study. Since colorectal cancer in Sweden is mainly performed through a midline incision, we think that this data is a good measurement of the complications and risk factors associated with the midline incision, and that is also why we choose to exclude laparoscopic procedures from this study.

The present study, however, has some limitations. The group of patients undergoing colorectal surgery is a heterogeneous one. Although the majority of the open procedures in this study were performed via a midline incision, there may have been other approaches used, especially the transverse incision. Furthermore, the length of incision varies. Data from primary care are not included in the registers, which may lead to underestimation of hernia incidence. One could also argue that this exclusion of hernias diagnosed in primary care and not referred to the surgeon gives us an estimate of clinically significant incisional hernias. Furthermore, patients with a hernia but deemed not fit for surgery at the preoperative assessment are missing in this analysis, thereby selecting patients in whom the risk for incisional hernia is probably lower. Indeed, we did not find any association between comorbidity and risk for incisional hernia, though this is probably explained by the reluctance to reoperate on high-risk patients, resulting in an under-reporting of incisional hernias. This could also explain why the incidence of incisional hernia was higher in patients younger than 70 years. Gender as a risk factor is controversial, and a similar recent study by Heyon Seo et al. finds that the female gender should be a risk factor [6]. It has also previously been shown that not high BMI but visceral obesity is a risk factor for incisional hernia [7]. The higher risk for men in our study could be an effect of visceral obesity, but further studies have to be done in order to accept or reject the role of gender.

In this study, the cumulative incidence of incisional hernia was 5.3%, which is close to that in other recent studies [4, 7] but lower than that in older studies [8]. Although the overall risk for incisional hernia is low, there are patients at risk that would benefit from prophylactic measures aiming to prevent incisional hernia. This study suggests that men, patients younger than 70 years, patients with BMI > 30 and patients undergoing procedures longer than 3 h could benefit from such prophylactic measures. Furthermore, we confirm that postoperative wound complication is a risk factor for incisional hernia and suggest that active measures to avoid wound complications will reduce the incidence of hernia.

As a rule, measures aiming at prevention of incisional hernia do not require large resources [1]. Nevertheless, the incidence of incisional hernia has remained at a level exceeding 10% (2–20%) 5 years after surgery [1]. This high incidence may reflect failure to comply with updated guidelines or the tendency to neglect abdominal wall complication as an outcome measure following abdominal surgery in favour of others. Despite the fact that numerous studies have shown that incisional hernia and wound dehiscence are not inevitable complications following abdominal surgery, they still continue to occur after colorectal surgery. These complication rates may be expected to fall as more procedures are performed using the laparosopic technique [6].

In conclusion, the cumulative incidence of incisional hernias 5 years after surgery is at least 5%. Patients at high risk for developing an incisional hernia will certainly benefit from active prevention measures if these are implemented in routine surgical practice. Risk factors include the male gender, age < 70 years, obesity, long operation time and postoperative wound complications.

## References

[1] Millbourn D, Wimo A, Israelsson LA (2014). Cost analysis of the use of small stitches when closing midline abdominal incisions. Hernia.

[2] Fazekas B, Fazekas B, Hendricks J, Smart N, Arulampalam T (2016). The incidence of incisional hernias following ileostomy reversal in colorectal cancer patients treated with anterior resection. Ann R Coll Surg Engl.

[3] Basta MN, Mirzabeigi MN, Shubinets V, Kelz RR, Williams NN, Fischer JP (2016). Predicting incisional hernia after bariatric surgery: a risk stratification model based upon 2161 operations. Surg Obes Relat Dis.

[4] Millbourn D, Cengiz Y, Israelsson LA (2009). Effect of stitch length on wound complications after closure of midline incisions: a randomized controlled trial. Arch Surg..

[5] Millbourn D, Cengiz Y, Israelsson LA (2011). Risk factors for wound complications in midline abdominal incisions related to the size of stitches. Hernia.

[6] Ludvigsson JF, Almqvist C, Bonamy AK, Ljung R, Michaëlsson K, Neovius M, Stephansson O, Ye W (2016). Registers of the Swedish total population and their use in medical research. Eur J Epidemiol.

[7] Seo GH, Choe EK, Park KJ, Chai YJ (2017) Incidence of clinically relevant incisional hernia after colon cancer surgery and its risk factors: a nationwide claims study. World J Surg. 10.1007/s00268-017-4256-4 10.1007/s00268-017-4256-4 28956105

[8] CT A, Rickles AS, Probst CP, Kelly KN, Deeb AP, Monson JR, Fleming FJ (2015). Visceral obesity, not elevated BMI, is strongly associated with incisional hernia after colorectal surgery. Dis Colon Rectum.

